# Discovery of a Potential Plasma Protein Biomarker Panel for Acute-on-Chronic Liver Failure Induced by Hepatitis B Virus

**DOI:** 10.3389/fphys.2017.01009

**Published:** 2017-12-06

**Authors:** Ni Zhou, Kuifeng Wang, Shanhua Fang, Xiaoyu Zhao, Tingting Huang, Huazhong Chen, Fei Yan, Yongzhi Tang, Hu Zhou, Jiansheng Zhu

**Affiliations:** ^1^Department of Infectious Diseases, Affiliated Taizhou Hospital of Wenzhou Medical University, Taizhou, China; ^2^E-Institute of Shanghai Municipal Education Committee, Shanghai University of Traditional Chinese Medicine, Shanghai, China; ^3^Department of Analytical Chemistry and CAS Key Laboratory of Receptor Research, Shanghai Institute of Materia Medica, Chinese Academy of Sciences, Shanghai, China

**Keywords:** HBV-ACLF, CHB, iTRAQ, proteomics, biomarker

## Abstract

Hepatitis B virus (HBV)-associated acute-on-chronic liver failure (HBV-ACLF), characterized by an acute deterioration of liver function in the patients with chronic hepatitis B (CHB), is lack of predicting biomarkers for prognosis. Plasma is an ideal sample for biomarker discovery due to inexpensive and minimally invasive sampling and good reproducibility. In this study, immuno-depletion of high-abundance plasma proteins followed by iTRAQ-based quantitative proteomic approach was employed to analyze plasma samples from 20 healthy control people, 20 CHB patients and 20 HBV-ACLF patients, respectively. As a result, a total of 427 proteins were identified from these samples, and 42 proteins were differentially expressed in HBV-ACLF patients as compared to both CHB patients and healthy controls. According to bioinformatics analysis results, 6 proteins related to immune response (MMR), inflammatory response (OPN, HPX), blood coagulation (ATIII) and lipid metabolism (APO-CII, GP73) were selected as biomarker candidates. Further ELISA analysis confirmed the significant up-regulation of GP73, MMR, OPN and down-regulation of ATIII, HPX, APO-CII in HBV-ACLF plasma samples (*p* < 0.01). Moreover, receiver operating characteristic (ROC) curve analysis revealed high diagnostic value of these candidates in assessing HBV-ACLF. In conclusion, present quantitative proteomic study identified 6 novel HBV-ACLF biomarker candidates and might provide fundamental information for development of HBV-ACLF biomarker.

## Introduction

Acute-on-chronic liver failure (ACLF) is increasingly recognized as an acute deterioration of liver function combining with liver and multi-organ failures in patients with pre-existing chronic liver disease, although there is no consensus about its definition (Bernal et al., 2015; Anand and Dhiman, 2016; Arroyo and Jalan, 2016). Hepatitis B virus (HBV) associated ACLF, as a subtype of ACLF, can develop at any stage in the progression of chronic hepatitis B (CHB) (Zamora Nava et al., 2014). It is estimated that about 70% of liver failure is caused by HBV infections in the Eastern countries (Sarin et al., 2009), and there are approximately 97 million people suffering from HBV infection in China alone (Cui and Jia, 2013). HBV-ACLF has a poor prognosis with high mortality rate (>70%), if emergency liver transplantation is not available (Marrero et al., 2003). Therefore, it is believed that predicting and stopping the progression of CHB to ACLF at an early stage may be the most effective strategy of reducing the mortality of patients with HBV-ACLF.

Despite a number of scoring systems such as Child-Pugh score have been used for diagnosis of end-stage liver diseases, not all of these scores are specifically designed for HBV-ACLF. So far, various biochemical molecules (e.g., Prealbumin, Serum ferritin), cytokines (e.g., Interleukin 17, Fibroleukin) and chemokines (e.g., Macrophage inflammatory protein-3α) have been evaluated to be novel indicators for HBV-ACLF as reviewed by Chen et al. (2015). Wan et al. (2015) performed a particle-enhanced immunonephelometry assay on serum samples from ACLF patients, and they found that the level of cystain C (CysC) was significantly higher in the ACLF with kidney failure group than those in the healthy controls and CHB patients. Their results suggested that CysC could be considered as a biomarker for renal dysfunction in ACLF patients. However, no protein biomarker has reached the clinical setting yet. Considering the complexity and heterogeneity of HBV-ACLF pathology, it has been suggested that integrated panel of biomarkers with specific and complementary functions rather than a single biomarker be useful in diagnosis of patients with HBV-ACLF.

Unbiased proteomic analysis of plasma samples holds the promise to discover clinically effective disease biomarkers. Plasma proteomics is an appealing concept in medicine due to inexpensive and minimally invasive sampling and good reproducibility (Harel et al., 2015). Plasma proteins comprise not only actual plasma proteins that maintain physiological homeostasis but also low abundance “leakage” proteins from damaged tissues, which may provide direct information about the pathology of disease and may serve as clinical biomarkers for diagnosis and treatment (Lv et al., 2007). Several studies have successfully applied this strategy to identify biomarkers of liver disease, including Hepatitis B (He et al., 2003), Hepatitis C (HCV) associated hepatic fibrosis (Yang et al., 2011) and HBV-associated hepatocellular carcinoma (HCC) (Niu et al., 2010).

With regard to HBV-ACLF, a study using matrix-assisted laser desorption/ionization time-of-flight (MALDI-TOF) mass spectrometry (MS) approach showed that protein profiling was markedly changed during the progression of CHB to liver failure and suggested that these dynamic changes can distinguish different stages of the CHB (Han et al., 2010). Another study employed two-dimensional gel electrophoresis (2-DE) MS/MS approach to compared serum samples collected from normal individuals, CHB patients and HBV-ACLF patients, which proposed that Alpha-1-acid glycoprotein (A1-AGF) might be a potential biomarker of ACLF diagnosis for CHB patients (Ren et al., 2010). Currently, with the advent of quantitative proteomic technology, isobaric tagging for relative and absolute quantitation (iTRAQ) technology makes it possible to quantify several proteins in a single experiment with improved accuracy and reproducibility of quantitation (Pierce et al., 2008). Using this technology, Peng et al. (2013) has identified total of 16 significantly differential proteins in serum from patients with CHB and patients with HBV-ACLF compared to healthy controls, and suggested five of those proteins were potentially associated with progression of hepatitis B and ACLF.

In this study, iTRAQ coupled with LC-MS/MS approach was utilized to construct the plasma proteome in healthy controls, patients with CHB and HBV-ACLF to explore disease-associated alterations of plasma proteins. In addition, we sought to validate several potential biomarkers that could distinguish ACLF from both CHB and healthy control by ELISA analysis and subsequent receiver operating characteristic (ROC) curves analysis. The six candidates identified in present study can aid clinical biomarker discovery for HBV-ACLF.

## Materials and methods

### Human plasma sample collection

Blood samples from healthy people (CON), patients with chronic hepatitis B (CHB) and patients with HBV induced acute-on-chronic liver failure (HBV-ACLF) were provided by Department of Infectious Diseases of Taizhou Hospital of Zhejiang Province, China (*n* = 20 per group). The study was approved by scientific ethics committee (Taizhou hospital of Zhejiang Province, China). Written informed consent was given from all participants and legal guardians before commencement of this study. The diagnoses of HBV-ACLF were based on criteria previously described (Sarin et al., 2009). Exclusion criteria included: pregnant or lactating women; liver cancer or suspected liver cancer; recent infection; use of immune-suppressive agents; anti-viral therapy, immune disease or malignant tumor; other types of hepatitis infection and HIV infection patients. Rejection criteria included: died within 7 days after enrollment, liver transplantation after enrollment. Information of clinical and demographic characteristics of patients with CHB, HBV-ACLF and healthy controls were shown in Table 1. HBV-ACLF samples were referred to as ACLF in the figure captions. The collected blood samples were then centrifuged at 3,000 rpm for 10 min at room temperature to remove any cells and debris. Twenty clarified plasma samples of each group were pooled into 4 samples that contained equal volume of 5 individual plasma samples from each group. As a result, a total of 60 samples were randomly pooled into 12 pooled samples.

**Table 1:** Clinical and demographic characteristics of subjects enrolled in this study.

**Variation**	**ACLF (*n* = 23)**	**CHB (*n* = 45)**	**CON (*n* = 20)**	***p*-value**
Age (year)	45.8 ± 13.3	40.9 ± 10.7	42.7 ± 10.9	0.250
Gender: male (%)	14 (60.9)	30 (66.7)	12 (60)	0.833
ALT (U/L)	427.2 ± 27.5	422.6 ± 16.4	30.2 ± 1.2	0.042
AST (U/L)	316.4 ± 13.5	189.3 ± 4.4	24.8 ± 0.4	<0.001
TB (μmol/L)	267.2 ± 3.8	37.6 ± 1.4	11.0 ± 0.2	<0.001
HBV-DNA (log_10_/mL)	5.4 ± 1.6	5.7 ± 1.4	ND	−
HBsAg: positive (%)	23 (100)	45 (100)	ND	−
HBeAg: positive (%)	16 (69.6)	33 (73.3)	ND	−

*ALT, alanine transaminase; AST, glutamic-oxalacetic transaminase; TB, total bilirubin; ND, not determined*.

### Plasma sample preparation for proteomic analysis

Since disease biomarkers in the plasma are usually covered by high-abundance proteins, and their signals are weak in the mass spectrum, removal of high-abundant proteins was performed using Agilent High-Capacity Human-14 Multiple Affinity Removal System (MARS Human-14, Agilent, USA) according to the manufacturer's instructions. Protein concentrations were determined by tryptophan fluorescence emission at 350 nm using an excitation wave length of 295 nm (Geiger et al., 2010). Removal effect was verified by Coomassie-stained gel. Then 100 μg of protein from each pooled sample was processed by the Filter Assisted Sample Preparation (FASP) method as previously described (Wiśniewski et al., 2010). Briefly, each sample was transferred to a 10 kDa filter (Millipore Corporation) and centrifuged at 14,000 g for 40 min at 20°C. Then, 200 μL of urea buffer (8 M urea, 0.1 M Tris-HCl, pH 8.5) was added and followed by another centrifugation at 15,000 g for 40 min. This step was repeated one more time. The concentrate was then mixed with 100 μL of 50 mM iodoacetamide (IAA) in urea buffer and incubated for an additional 40 min at room temperature in darkness. After that, IAA was removed by centrifugation at 14,000 g for 40 min. Next, the sample was diluted with 200 μL of urea buffer and centrifuged two more times. Then, 200 μL of 50 mM tetraethyl ammonium bromide (TEAB) was added and the sample was centrifuged at 14,000 g for 40 min. This step was repeated twice. Finally, samples were digested with trypsin (1:50, enzyme to protein in 50 mM TEAB) by incubating at 37°C for 16 h.

### iTRAQ labeling of plasma samples

Peptides were labeled with iTRAQ reagents according to the manufacturer's instructions (AB Sciex, Foster City, CA). To quantify 12 samples, 2 batches of 8-plex iTRAQ labeling experiment were performed, with a mixture of 12 samples in equal amount as a bridge for comparison among different batches. Each aliquot (50 μg of peptide equivalent) was reacted with one tube of iTRAQ reagent. After the sample was dissolved in 15 μL of 0.5 M TEAB solution, pH 8.5, the iTRAQ reagent was dissolved in 50 μL of isopropanol. The mixture was incubated at room temperature for 2 h. The 8-plex labeled samples in the same experiment branch was pooled together and lyophilized.

### High pH reverse phase fractionation (HPRP)

iTRAQ-labeled peptides mixture was fractionated using a Waters XBridge BEH130 C18 3.5 μm 2.1 × 150 mm column on a Agilent 1260 HPLC operating at 0.2 mL/min. Buffer A consisted of 10 mM ammonium formate and buffer B consisted of 10 mM ammonium formate with 90% acetonitrile; both buffers were adjusted to pH 10 with ammonium hydroxide as described previously (Wang et al., 2011). A CBS-B programed multifunction automatic fraction collecting instrument (Huxi instrument, Shanghai, China) was coupled to the HPLC and used to collect eluted peptides. A total of 28 fractions were collected for each peptides mixture, and then concatenated to 14 (pooling equal interval RPLC fractions). The fractions were dried for nano LC-MS/MS analysis.

### LC-MS/MS analysis

The reverse phase high-performance liquid chromatography (RP-HPLC) separation was achieved on the Easy nano-LC system (Thermo Fisher Scientific) using a self-packed column (75 μm × 150 mm; 3 μm ReproSil-Pur C18 beads, 120 Å, Dr. Maisch GmbH, Ammerbuch, Germany) at a flow rate of 300 nL/min. The mobile phase A of RP-HPLC was 0.1% formic acid in water, and B was 0.1% formic acid in acetonitrile. The peptides were eluted using a gradient (2–90% mobile phase B) over a 90 min period into a nano-ESI Orbitrap Elite mass spectrometer (Thermo Fisher Scientific). The mass spectrometer was operated in data-dependent mode with each full MS scan (m/z 300–1,500) followed by MS/MS for the 12 most intense ions with the parameters: ≥ +2 precursor ion charge, 2 Da precursor ion isolation window, 80 first mass and 38 normalized collision energy of HCD. Dynamic Exclusion™ was set for 30 s. The full mass and the subsequent MS/MS analyses were scanned in the Orbitrap analyzer with *R* = 60,000 and *R* = 15,000, respectively.

### Database searching and analysis

Data were processed by search against the UniProt/SwissProt Human database (IPI.human.v3.87) using Maxquant (version 1.5.1.0), with default settings including the allowance of one missed cleavage and 8-plex iTRAQ fixed modifications. Minimum 7 amino acids for peptide, >2 peptides were required per protein. For peptide and protein identification, false discovery rate (FDR) was set to 1%. iTRAQ reporter ion intensity were used for quantification. By setting the median of intensity for each channel to equal and matching the distributions of each treatment iTRAQ reporter group (114, 115, 116, 118, 119, and 121) to those of the control iTRAQ reporter group (113, which corresponded to the mixture sample), we able to make consistent comparisons across different samples obtained from different iTRAQ 8-plex experiments. The ratio was restored to the intensity by multiplying the median of intensity of the first channel 113 (MIX1).

### Bioinformatics analysis

Functional enrichment analysis of Gene Ontology (GO) of biological process, molecular function, and cellular component was performed using DAVID Bioinformatics Resources version 6.7. The protein-protein interaction (PPI) network analysis of differentially expressed proteins was performed using STRING (https://www.string-db.org/). And the PPI network was further processed by Cytoscape software.

### Elisa assay

The expression levels of selected biomarkers were measured in plasma samples from 20 healthy controls, 45 CHB patients and 23 HBV-ACLF patients using ELISA quantitation kits (APO-CII, GP73, OPN, MMR, HPX purchased from RayBiotech; ATIII purchased from R&D systems, UK). The experimental methods were carried out according to the manufacturer's instructions.

### Evaluation of the diagnostic accuracy

Mathematical models for separation of HBV-ACLF from CHB patients were performed on ELISA results of 6 candidate biomarkers using SPSS 19.0 software (Chicago, IL, USA). The diagnostic score of CHB patient was set as “0,” while that of ALCF patient was set as “1.” The forward stepwise multivariate regression analysis was conducted to determine which proteins should be included or excluded from the diagnostic model. The global performances of the model and individual biomarkers were evaluated by constructing receiver operating characteristic (ROC) curves and calculating the area under the curve (AUC) values.

### Statistical analysis

One way analysis of variance (ANOVA) and Tukey's honestly significant difference (HSD) test was performed with language R. *p*-value <0.05 was defined as statistically significant. Clinical chemistry data are expressed as mean ± SEM. Hierarchical clustering of proteins was performed on logarithmized data, using Euclidean distances and Ward clustering method using Package of “pheatmap” in language R. Correlation between samples was analyzed using Spearman's rank correlation coefficient.

## Results

### Identification of significantly changed proteins in CHB and HBV-ACLF groups

**Figure 1:**
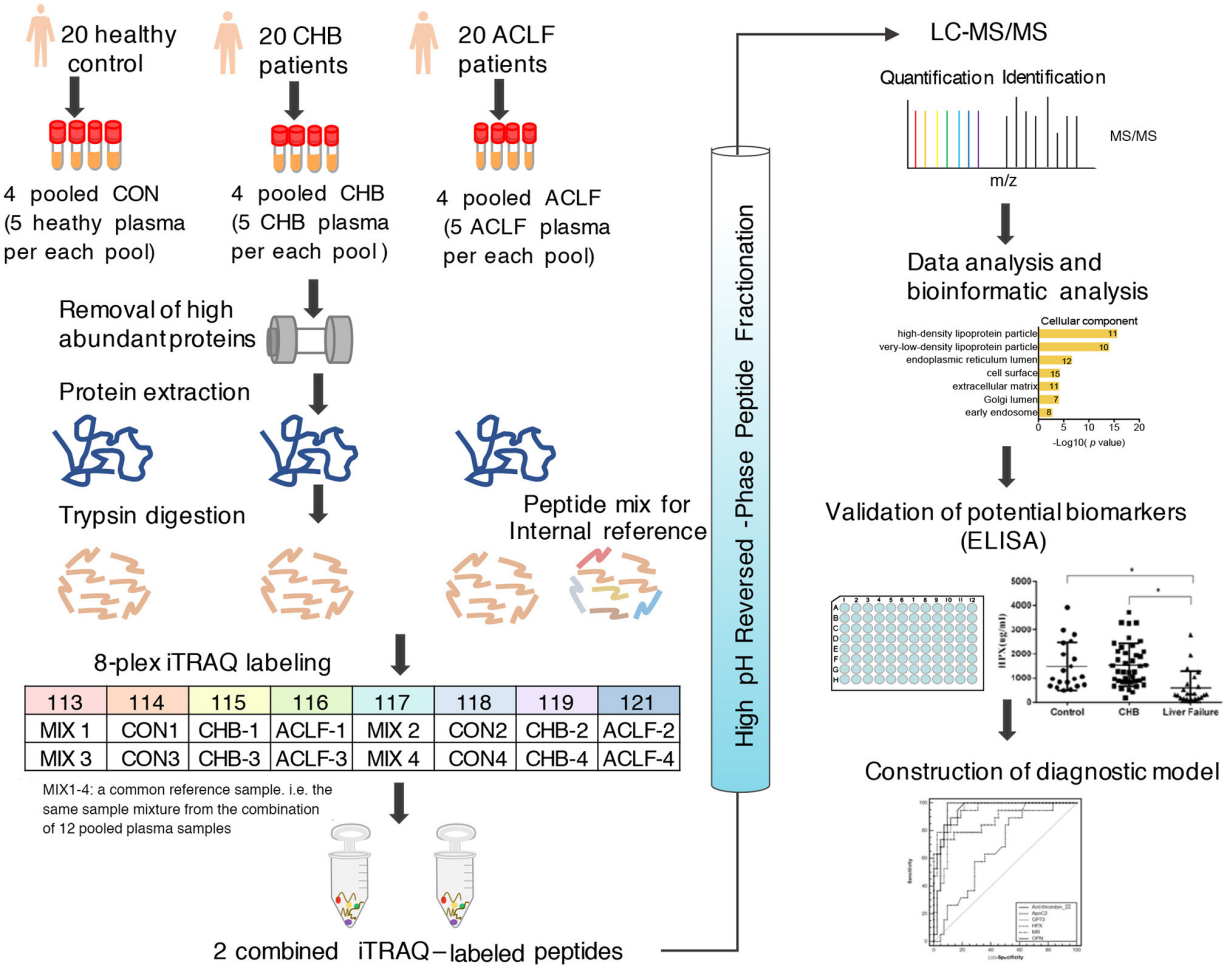
Workflow chart of the proteomic study. Twelve pooled plasma samples (pooled from *n* = 5) from 20 healthy controls, 20 CHB patients and 20 ACLF patients were subjected to removal of high abundant proteins. Then equal amounts of proteins from each sample were digested with trypsin. Resultant peptides were processed 8-plex iTRAQ labeling, HPRP fractionation and subsequent LC-MS/MS analysis. Bioinformatics analysis was performed using Uniprot and STRING database. The plasma levels of candidate proteins were further verified by ELISA assay and diagnostic value of these biomarkers were assessed by forward stepwise logistic regression analysis and ROC curve analysis.

In this study, plasma samples from healthy control, CHB and HBV-ACLF patients were subjected to LC-MS/MS analysis following removal of high abundance protein, FASP preparation, tryptic digestion and iTRAQ labeling. The experimental workflow is illustrated in Figure 1. iTRAQ 113 and 117 were used to label the mixture of all samples as a reference pool in different sets, thus allowing for cross-set comparison (Song et al., 2008).

**Figure 2:**
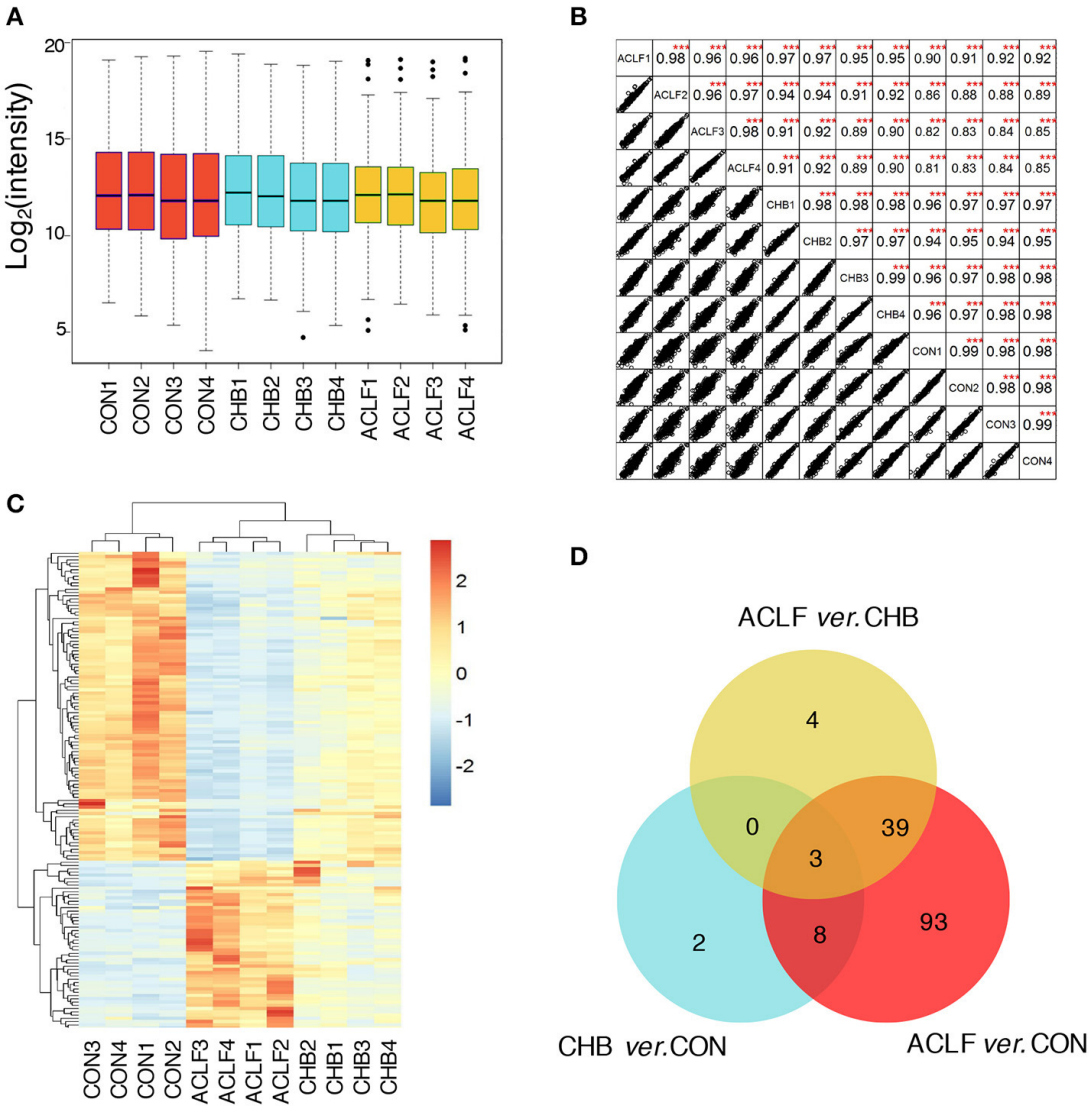
Identification of total proteins and differentially expressed proteins. **(A)** Box plots of log_2_ protein intensity average for each sample. **(B)** Correlation analysis between each two samples. Rows and columns represent samples, and each square shows the correlation coefficients between two samples. ^***^p < 0.001 comparing intensity of each two samples. **(C)** Heatmap of the significantly changed proteins. Rows represent proteins and columns represent different samples. Color of each cell represents expression change of proteins, red is increased and blue is decreased relative to control group. **(D)** Venn diagram shows the overlap of differential proteins between comparison of each two groups.

With the false discovery rate (FDR) <1%, 397 and 396 proteins were identified in the 8-plex iTRAQ data set 1 and 2, respectively, resulting in a total of 427 proteins identified (Table S1). Of which, 364 non-redundant proteins were commonly identified across all samples by these two iTRAQ experiments. The quality of the proteomic dataset and instrumental reproducibility was evaluated. As shown in Figure 2A, the box plot analysis showed that the log_2_ protein intensity medians of all 12 pooled samples were about 1.25, almost at the same levels across all the samples, suggesting that there were no biases toward any samples. In addition, correlation analysis was performed on intensities between biological replicates inside each cohort or between different cohorts. Figure 2B showed that all of correlation coefficients between each two samples were higher than 0.86, demonstrating good reproducibility of biological replicates. Taken together, these results suggest that the iTRAQ-MS/MS analysis yielded a high quality reproducible dataset.

Filtering the iTRAQ data set using criteria of *p*-value < 0.05 and fold change >2, we identified a total of 149 significantly changed proteins through comparing each two groups. A full list of all significantly differential proteins is given in Table S2.

A heatmap analysis of 149 differential proteins using an unsupervised hierarchical clustering and correlation distance metric was generated to depict the change of expression level in different groups (Figure 2C). As the dendrogram indicated, CON, CHB and HBV-ACLF group samples formed three distinct clusters and the individuals within each group displayed the closest relationship.

Venn diagrams displayed unique and overlapping differential proteins in CHB and HBV-ACLF as compared to CON. As shown in Figure 2D, 3 overlapping differential proteins were identified by comparison of each two groups. There were 143 proteins differentially expressed between HBV-ACLF and CON, of which 42 were significantly changed when HBV-ACLF was compared to both CON and CHB. There were only 13 proteins differentially expressed between CHB and CON.

### Bioinformatics analysis of differentially expressed proteins

**Figure 3:**
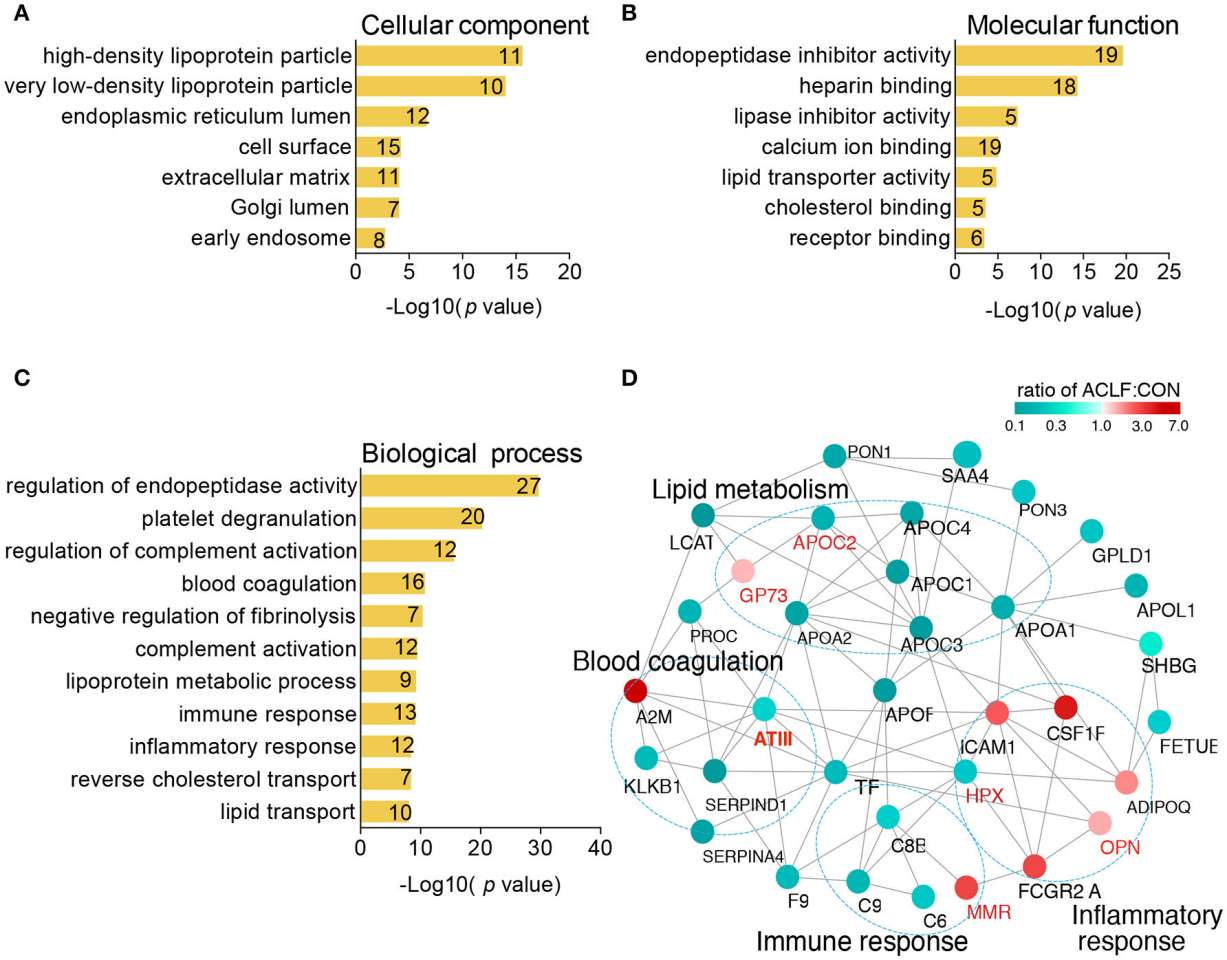
Bioinformatics analysis of differentially expressed proteins. All of 147 proteins were functionally annotated in according to their cellular component **(A)**, molecular function **(B)**, and biological process **(C)**. The x axis represent the negative log of *p*-value. Digits mentioned inside each bar represent the number of proteins involved in each GO term. **(D)** Protein-protein interaction analysis of 42 differential proteins between CHB and HBV-ACLF using STRING database. Interactions between two proteins were indicated with gray edges. Color of node indicates fold change in ACLF. Green represents down-regulated protein and red represents up-regulated protein. Manual functional annotations based on GO analysis were shown.

To understand biological significance regarding to differentially expressed proteins in HBV-ACLF patients, the cellular component, molecular function and biological process of the 143 proteins were explored by Gene Ontology (GO) annotation (Table S3). In the cellular component category of GO, the most over-represented term is high-density lipoprotein particle (Figure 3A) and the most significant molecular function is endopeptidase inhibitor activity (Figure 3B). The top 3 biological processes terms were regulation of endopeptidase activity, platelet degranulation and regulation of complement activation. Other important biological processes such as regulation of fibrinolysis, complement activation, immune response and inflammatory response were also over-represented (Figure 3C).

To understand functional relationship among the 42 differential proteins between CHB and HBV-ACLF groups, protein protein interaction (PPI) network based on STRING action scores was illustrated. The annotations of biological processes based on GO analysis were also indicated in this view. PPI analysis showed a complex network with several distinct biological subgroups that contained highly connected proteins. As shown in Figure 3D, proteins involved in immune response, inflammatory response, blood coagulation and lipid metabolism were highly connected with each other, indicating that functional network of these processes contribute to HBV-ACLF pathophysiology. Based on promising reports from literature, 6 proteins antithrombin-III (ATIII), mannose receptor (MMR), golgi membrane protein 1 (GP73), osteopontin (OPN), apolipoprotein CII (APO-CII), hemopexin (HPX) involved in biological processes mentioned above were selected for further verification.

### Evaluation of six selected proteins as biomarker candidates

**Figure 4:**
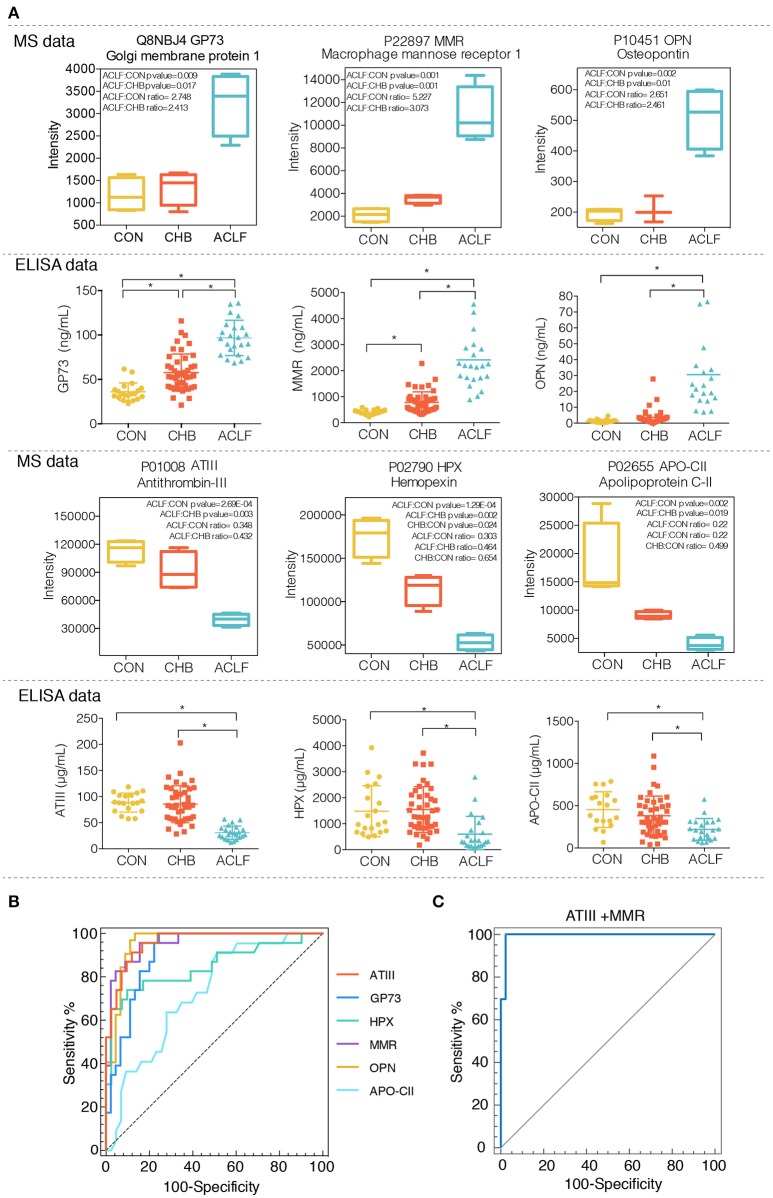
Evaluation of plasma levels of 6 candidate proteins in healthy controls, CHB patients and HBV-ACLF patients using ELISA assay. **(A)** Plasma levels of six candidates (ATIII, HPX, APO-CII, GP73, MMR, and OPN) in different groups were analysis by ELISA assay. Median values were shown with a horizontal line. ^*^p < 0.01, Upper panel indicates protein intensity of each candidate obtained from iTRAQ-proteomic analysis. **(B)** ROC curve analysis of the 6 individual biomarkers. **(C)** ROC curve analysis of the combination of ATIII and MMR.

To verify whether alterations of 6 selected candidates are reliably presented in clinical samples, we performed an ELISA assay to measure protein levels in plasma samples from healthy controls (CON, *n* = 20), CHB patients (CHB, *n* = 45), HBV-ACLF patients (ACLF, *n* = 23). The results showed significant elevation of GP73, MMR, and OPN (*p* < 0.01) and significant reduction of ATIII, HPX, APO-CII expression levels (*p* < 0.01) in the HBV-ACLF group as compared to both CHB and CON groups. In addition, significant differences in GP73 and MMR levels were also observed between the CHB and CON groups (*p* < 0.01). These results are consistent with the data obtained from the proteomic studies (Figure 4A).

Subsequently, the diagnostic values of 6 candidates were analyzed by forward stepwise multivariate regression. The result showed that MMR and ATIII were included in this logistic regression model as below (e is the mathematical constant and base value of natural logarithms):

$$\begin{array}{l} P={\text{e}}^{-0.0496+0.006\text{MMR}-0.176\text{ATIII}}/1+{\text{e}}^{-0.0496+0.006\text{MMR}-0.176\text{ATIII}} \end{array}$$

Odds ratios of MMR and ATIII in the diagnostic model were 1.006 and 0.839 respectively. Furthermore, receiver operating characteristic (ROC) curve was exploited based on the results of the area under the curve (AUC), sensitivity and specificity. Figures 4B,C and Table 2 showed the results of ROC analysis of individual biomarkers and the combined biomarker model for discriminating liver failure patients from CHB patients. The AUC of the combined biomarker model ATIII+MMR was 0.993, higher than any other individual biomarkers, indicating the combination of ATIII and MMR can effectively discriminate the HBV-ACLF patients from CHB patients.

**Table 2:** ROC analysis of individual biomarkers and combined diagnostic model.

**Proteins**	**AUC**	**95% CI**	**Sensitivity (%)**	**Specificity (%)**
GP73	0.91	0.81–0.96	100.00	75.56
HPX	0.86	0.75–0.93	73.91	90.91
MMR	0.96	0.88–0.99	82.61	95.56
OPN	0.96	0.87–0.99	100.00	88.64
ATIII	0.96	0.89–0.99	91.30	88.89
APO-CII	0.72	0.60–0.82	90.91	50.00
MMR+ATIII	0.99	0.93–1.00	100.00	97.78

## Discussion

To our knowledge, there are two representative ACLF definitions proposed by the Asia-Pacific Association for the Study of the Liver (APASL) and the American Association for the Study of Liver Disease and the European Association for the Study of the Liver (AASLD/EASL) (Kim and Kim, 2013). The APASL focused on the occurrence of complication such as ascites and encephalopathy within 4 weeks in patients with chronic liver disease (Sarin et al., 2009), whereas the other one emphasized the occurrence of multi-organ failure and 3 months mortality (Olson and Kamath, 2011). However, most researchers agree that the concept of ACLF should include: acute deterioration of pre-existing chronic liver disease, multi-system organ failure and with a mortality ≥15% at day 28 (Kim and Kim, 2013; Blasco-Algora et al., 2015). Unfortunately, there is a lack of biomarker highly sensitive and minimally invasive to predict ACLF in CHB patients. In this study, plasma proteome profiling of healthy controls, CHB patients and HBV-ACLF patients was established by iTRAQ-based proteomic analysis, aiming to search novel diagnostic biomarkers of HBV-ACLF. We identified 6 candidates with strong biological relevance to HBV-ACLF pathogenesis and further confirmed their change of plasma levels in 68 subjects using ELISA assay.

Demographic information exhibited that approximately 60% patients are male in either CHB or ACLF group as shown in Table 1. This result was consistent with the study from Rifai et al. (2012). They found that significantly more males than females underwent liver transplantation for CHB. There may be a gender difference with more men susceptible to HBV infection and developing to end-stage liver disease, which could be attribute to sex hormone effects on HBV transcription and immune response to HBV infection (Wang et al., 2015).

Individual variations among patients make a big challenge for applications of conventional proteomics. This issue has been addressed in plasma proteomic studies (Zhou et al., 2012). In the present study, particularly, each 5 plasma samples in the same group were randomly pooled to minimize the individual variations (Schisterman and Vexler, 2008). Plasma has been widely used in proteomic study for biomarker discover. However, the large dynamic range of protein concentrations in plasma samples exceeds the analytical capabilities of traditional proteomic methods, making those lower abundance plasma proteins undetectable (Pernemalm and Lehtiö, 2014). Therefore, we firstly conducted removal of high-abundance proteins (such as Albumin and IgG) using immune affinity-based depletion method to improve depth of detection in plasma sample. In doing so, a total of 427 proteins were identified across all samples. We found that more extensive molecular response was occurred in progression of ACLF (143 altered proteins as compared to healthy control) than in that of CHB (13 altered proteins as compared to healthy control).

Accurate diagnostic prediction is critical for distinguishing CHB patients who require transplantation from those who will survive following intensive medical care alone. Current Venn diagram revealed that expression of 42 proteins were changed significantly when HBV-ACLF group were compared with both healthy control and CHB groups, indicating these proteins may be helpful in identifying biomarkers for discriminating HBV-ACLF from CHB patients. Analysis of the protein-protein interaction of 42 proteins revealed that these proteins connected each other to regulate distinct biological process, including immune response, inflammatory response, blood coagulation, and lipid metabolic process.

It is well accepted that ACLF is an exaggerated systemic inflammatory response in context of immune dysregulation. The inflammation may also result in the unbalance of pro-thrombotic and anti-thrombotic states that may be manifested by either bleeding or thrombotic complications (Blasco-Algora et al., 2015). Since these biological processes are closely related to ACLF pathology, we considered 6 proteins (MMR, OPN, HPX, GP73, ATIII, and APO-CII) involved in these processes as potential biomarkers for diagnosis of HBV-ACLF and the clinical relevance of these proteins was further confirmed by ELISA assay. Subsequent ROC analysis indicated that these candidates, especially combination of MMR and ATIII, have good sensitivity and specificity in predicting HBV-ACLF.

Mannose receptor (MMR) locates on the surface of various cell types such as macrophages and dendritic cells (Martinez-Pomares, 2012). As a pattern recognition receptor, MMR binds and internalizes the glycoproteins from various pathogens (e.g., virus, bacteria and parasites) (Stahl and Ezekowitz, 1998), thus playing an important role in innate and adaptive immune response (Apostolopoulos and McKenzie, 2001). Another function of the MMR is to eliminate inflammatory agents released into the circulation during the inflammatory response (Lee et al., 2002). It has been reported that the concentration of soluble MMR (sMR) in serum from CHC patients with cirrhosis was higher than that with mild hepatic fibrosis patients (Andersen et al., 2014). Similarly, our study showed that levels of plasma MMR in HBV-ACLF patients were higher than CHB patients and healthy controls. It can be speculated that MMR-mediated immune and inflammatory response was dramatically triggered in context of HBV-ACLF.

Osteopontin (OPN), as a phosphorylated integrin-binding protein, has been implicated in many distinct pathophysiological processes including wound healing, bone turnover and tumorigenesis. Particularly, its roles in immune response and inflammation have been extensively studied (Rittling and Singh, 2015). OPN contributes to development of immune-mediated and inflammatory disease by promoting inflammatory cells recruitment (Apte et al., 2005), enhancing B cell proliferation (Wang and Denhardt, 2008) and suppressing apoptosis of immune cells (Denhardt et al., 2001). Several studies have shown that expression level of OPN was positively associated with CHB, CHC, alcoholic liver disease, fibrosis and HCC (Nagoshi, 2014; Fouad et al., 2015; Duarte-Salles et al., 2016). Recent studies reported remarkable elevation of serum OPN concentration in fulminant hepatic failure (FHF) patients and acute liver failure patients (Arai et al., 2006; Srungaram et al., 2015). Consistent with these findings, this study showed that level of plasma OPN was significantly elevated in HBV-ACLF groups as compared to CHB and healthy control groups, and this up-regulation of OPN may aggravate hepatic inflammation of CHB patients in progression to ACLF.

Hemopexin (HPX), as an acute phase glycoprotein, can bind heme with high affinity (Paoli et al., 1999), and the resultant Heme-HPX complex can be taken up by liver, protecting the body against free heme-induced oxidative damage (Hvidberg et al., 2005). Recent study reported anti-inflammatory function of HPX through its ability to regulate the pro-inflammatory cytokines and infiltration of Th17 cell (Liang et al., 2009). Xu et al. (2014) concluded that serum HPX concentration is negatively associated with severity of rat acute rejection after liver allograft. In addition, decrease of of HPX level was also observed in rat model of liver fibrosis induced by carbon tetrachloride (CCl_4_) (Zhang et al., 2015). In present study, plasma HPX level was significantly reduced in HBV-ACLF patients compared to CHB patients and healthy controls. Low HPX level may be attribute to impaired function of hepatocyte that is principal site of HPX synthesis, and decreased HPX may further aggravate liver damage. However, the role of HPX in pathogenesis of HBV-ACLF remains elusive to contradictory results reported by Lu et al. (2010), where up-regulation of HPX level was observed in plasma sample from HBV infected patients with liver fibrosis.

Golgi membrane protein 1 (GP73), as a type II Golgi membrane protein with unknown function, mainly presents in biliary epithelial cell and is rarely expressed in normal hepatocytes (Ba et al., 2012). However, serum GP73 levels are dramatically elevated in context of various types of liver disease such as viral infection (HBV, HCV), alcohol-induced liver disease (Kladney et al., 2002), cirrhosis (Iftikhar et al., 2004), or HCC (Gao et al., 2015; Sai et al., 2015; Zhang et al., 2016). Wei et al. (2014) revealed that expression level of serum GP73 was significantly up-regulated in patient with HBV-ACLF compared to HCC patients, CHB patients, and healthy controls, supporting our present result. Biological significance of GP73 elevation requires further study.

One of the typical clinical characteristics of liver failure is coagulation dysfunction because of the dysregulated production of coagulation factors and anti-coagulation factors. Antithrombin-III (ATIII), exclusively synthesized by hepatocytes, is a natural anticoagulant that inactivates several enzymes of coagulation system (Castelino and Salem, 1997). It was reported that ATIII levels are reduced in various liver disorders, such as cirrhosis, hepatitis and the fatty liver of pregnancy (Castelino and Salem, 1997). Tischendorf et al. (2016) suggested that reduced activity of ATIII was independent predictors of hepatic encephalopathy in patients with liver cirrhosis. Evaluating 158 HCC patients subjected to hepatectomy, Mizuguchi et al. (2012) demonstrated the decrease of serum ATIII as a useful predictor for postoperative liver dysfunction post hepatectomy. Similar conclusion was also yielded by Kuroda et al. (2015). In line with these findings, plasma ATIII level was significantly reduced in HBV-ACLF patients group.

Liver is the primary site of production for apolipoproteins that is responsible for the maintenance of lipoproteins and lipid metabolism (Bell, 1979). Apolipoprotein CII (APO-CII), together with APO-CI and APO-CIII are constituents of chylomicrons, very low-density lipoprotein (VLDL), and high-density lipoprotein (HDL) in blood circulation. APO-CII regulates triglyceride metabolism through interact with lipoprotein lipase (LPL), a enzyme for hydrolysis and clearance of triglycerides from VLDL and chylomicrons (Jong et al., 1999). However, both an excess and a lack of APO-CII inhibit LPL activity and thus result in hypertriglyceridemia (Kei et al., 2012). Song et al. (2012) concluded that reduced serum APO-CII and APO-CIII were associated with aberrant biliary cycle, and considered APO-CII and APO-CIII as potential biomarker for diagnosis of biliary atresia. Trieb et al. (2016) found that decrease of serum APO-CIII level was associated with cirrhosis mortality. However, the relationship between APO-CII and liver damage such as ACLF has not been reported. Previous study has suggested that APO-CII levels would not be affected in most patients with liver disease, despite a down-regulation of APO-CIII levels (Koga et al., 1984). Our study showed that the plasma concentration of APO-CII was reduced in HBV-ACLF patients compared to both CHB patients and healthy controls, which may reflect impairment of lipid metabolism in HBV-ACLF disease.

According to the previous proteomic study reported by Peng et al. (2013), there were 16 proteins differentially expressed in CHB and ACLF patients. We did the comparison of our differentially expressed proteins with their 16 proteins, and three proteins were overlapped, including vitronectin (VTN), C-reactive protein (CRP) and platelet factor 4 (PF4). In their study, vitronectin (VTN) showed 1.23- and 2.14-fold down-regulation in CHB and ACLF patients, respectively, and this protein was also down-regulated in our dataset with the ratio of CHB/CON = 0.73 in CHB patients and the ratio of ACLF/CON = 0.48 in ACLF patients (Table S2). As a cell adhesion and spreading factor found in serum and tissues, VTN was reported that its plasma level dramatically decreased in chronic liver disease (Tomihira, 1991; Kobayashi et al., 1994). In Peng et al.'s study, pro-inflammatory protein C-reactive protein (CRP) was 2.46-fold down-regulated and 4.59-fold up-regulated in CHB and ACLF patients, respectively, and our data demonstrated that CRP was up-regulated with the ratio of CHB/CON = 1.97 in CHB patients and the ratio of ACLF/CON = 5.43 in ACLF patients. Platelet factor 4 (PF4) was down-regulated with 1.15- and 1.87-fold in CHB and ACLF patients respectively, and our data is consistent with theirs with the ratio of CHB/CON = 0.21 in CHB patients and the ratio of ACLF/CON = 0.12 in ACLF patients. Thus, the similar tendency of these three proteins between Peng et al.'s study and ours suggested that the potential clinical application of these proteins for HBV-ACLF diagnosis can be further investigated.

In summary, this study employed an iTRAQ-based quantitative proteomic approach to identify plasma biomarkers for HBV-ACLF diagnosis. Based on protein-protein interaction analysis, we focused on 6 differentially expressed proteins involved in inflammation, immune response, blood coagulation and lipid metabolism. And the following ELISA analysis of plasma samples from patient cohorts further confirmed the up-regulation of GP73, MMR, OPN and the down-regulation of ATIII, HPX, APO-CII in HBV-ACLF patients. These proteins were involved in the key pathological processes on acute occurrence of complication or multi-organ failure in the progression of ACLF. So we believed that these proteins can be considered as the potential biomarkers for HBV-ACLF diagnosis. However, more confirmatory studies are required with hope that theses candidate biomarkers can be applied to routine clinical practice.

## Author contributions

NZ, KW, SF, HZ and JZ designed the experiments and wrote the manuscript. NZ, KW and SF performed the experiments with the support and help from FY and YT. NZ and XZ performed the clinic sample collection and ELISA experiments and statistical data analysis with the assistance of TH and HC. All authors critically reviewed content and approved final version for publication.

### Conflict of interest statement

The authors declare that the research was conducted in the absence of any commercial or financial relationships that could be construed as a potential conflict of interest.

## Abbreviations

HBV-ACLF: HBV induced acute-on-chronic liver failure
MMR: mannose receptor
OPN: osteopontin
HPX: hemopexin
GP73: golgi membrane protein 1
ATIII: antithrombin-III
APO-CII: apolipoprotein CII
CHB: chronic hepatitis B
CHC: Chronic hepatitis C.
